# Machine learning in neurosurgery: a global survey

**DOI:** 10.1007/s00701-020-04532-1

**Published:** 2020-08-18

**Authors:** Victor E. Staartjes, Vittorio Stumpo, Julius M. Kernbach, Anita M. Klukowska, Pravesh S. Gadjradj, Marc L. Schröder, Anand Veeravagu, Martin N. Stienen, Christiaan H. B. van Niftrik, Carlo Serra, Luca Regli

**Affiliations:** 1 Machine Intelligence in Clinical Neuroscience (MICN) Lab, Department of Neurosurgery, Clinical Neuroscience Center, University Hospital Zurich, University of Zurich, Frauenklinikstrasse 10, 8091 Zurich, Switzerland; 2 Amsterdam UMC, Vrije Universiteit Amsterdam, Neurosurgery, Amsterdam Movement Sciences, Amsterdam, The Netherlands; 3 Department of Neurosurgery, Bergman Clinics, Amsterdam, The Netherlands; 4 Università Cattolica del Sacro Cuore, Rome, Italy; 5 Department of Neurosurgery, RWTH Aachen University Hospital, Aachen, Germany; 6 School of Medicine, University of Nottingham, Nottingham, UK; 7 Department of Neurosurgery, Leiden University Medical Centre, Leiden, The Netherlands; 8 Department of Neurosurgery, Erasmus MC, University Medical Centre, Rotterdam, The Netherlands; 9 Neurosurgery AI Lab, Department of Neurosurgery, Stanford University, Stanford, CA USA

**Keywords:** Machine learning, Artificial intelligence, Technology, Neurosurgery, Global, Worldwide survey

## Abstract

**Background:**

Recent technological advances have led to the development and implementation of machine learning (ML) in various disciplines, including neurosurgery. Our goal was to conduct a comprehensive survey of neurosurgeons to assess the acceptance of and attitudes toward ML in neurosurgical practice and to identify factors associated with its use.

**Methods:**

The online survey consisted of nine or ten mandatory questions and was distributed in February and March 2019 through the European Association of Neurosurgical Societies (EANS) and the Congress of Neurosurgeons (CNS).

**Results:**

Out of 7280 neurosurgeons who received the survey, we received 362 responses, with a response rate of 5%, mainly in Europe and North America. In total, 103 neurosurgeons (28.5%) reported using ML in their clinical practice, and 31.1% in research. Adoption rates of ML were relatively evenly distributed, with 25.6% for North America, 30.9% for Europe, 33.3% for Latin America and the Middle East, 44.4% for Asia and Pacific and 100% for Africa with only two responses. No predictors of clinical ML use were identified, although academic settings and subspecialties neuro-oncology, functional, trauma and epilepsy predicted use of ML in research. The most common applications were for predicting outcomes and complications, as well as interpretation of imaging.

**Conclusions:**

This report provides a global overview of the neurosurgical applications of ML. A relevant proportion of the surveyed neurosurgeons reported clinical experience with ML algorithms. Future studies should aim to clarify the role and potential benefits of ML in neurosurgery and to reconcile these potential advantages with bioethical considerations.

## Introduction

Recent years have witnessed the rise of machine learning applications in the scientific literature, both in basic science and clinical medicine [18, 26]. Neurosurgical practice has always relied on the individual experience of surgeons to carefully balance surgical indications, operative risk and expected outcome [30]. The advent of evidence-based medicine has framed the surgical decision-making process into guidelines based on the results of high-quality data, and of randomized controlled clinical trials—not devoid of several flaws in design themselves [19]. This approach, despite remaining the gold standard, is limited by the oversimplification of patients’ individual characteristics that often do not allow patient-specific analytics. With the exponential growth of data in the era of big data, it is increasingly important to provide clinicians with tools for integrating this individual patient data into reliable prediction models. The latter primarily aims to enhance the surgical decision-making processes and potentially improve outcomes, but predictive analytics furthermore harbour the potential to reduce unnecessary health-care costs [21, 29, 31, 34, 36, 37, 41].

It is often difficult for clinicians to integrate the many described risk factors and outcome predictors into a single workable prognosis [3]. Neurosurgical research and clinical practice is ideal for the application of machine learning (ML), which harbours the potential for predictive analytics to integrate all relevant patient factors in a way that is often too complex for natural intelligence [28, 40]. Moreover, ML can be used to extract deep features from data such as radiological and histological images, or genomic data [16, 38–40, 43].

At present, the neurosurgical literature is increasingly focusing on substituting traditional statistical models with more complex ML models with the aim of improving predictive power [29, 31]. For example, ML has been used in neurosurgery to predict post-operative satisfaction [2], early post-operative complications [41] or cerebrospinal fluid leaks [37]. Despite this encouraging trend and the presence of recent publications reviewing the large range of publications on ML in neurosurgery [28–30], data on the worldwide adoption and perception of ML in our specialty are currently lacking. Our aim was to carry out a worldwide survey among neurosurgeons to assess the adoption of ML algorithms into neurosurgical clinical practice and research and to identify factors associated with their use.

## Materials and methods

### Sample population

The survey was distributed via the European Association of the Neurosurgical Societies (EANS) and Congress of Neurological Surgeons (CNS) in January, February and March 2019. The EANS is the professional organization that represents European neurosurgeons. An email invitation was sent through the EANS newsletter on January 28, 2019. Furthermore, the membership database of the CNS was searched for email addresses of active members and congress attendants. The CNS is a professional, US-based (US) organization, that represents neurosurgeons worldwide. At the time of the search, the database contained 9007 members from all continents. A total of 7280 neurosurgeons had functioning email addresses and were recipients of the survey. The survey was hosted by SurveyMonkey (San Mateo, CA, USA) and sent by email alongside an invitation letter. Reminders were sent after 2 and 4 weeks to non-responders to increase the response rate. To limit answers to unique site visitors, each email address was only allowed to fill in the survey once. All answers were captured anonymously. No incentives were provided.

### Survey content

The online survey was made up of nine or ten compulsory questions, depending on the participants’ choice of whether they had or had not used ML in their neurosurgical practice. A complete overview of survey questions and response options is provided in Table 1. The order in which potential reasons for use/non-use were displayed was randomized to avoid systematic bias. The definition of ML applications that were provided within the survey was: “Any form of artificial intelligence (AI)–based or algorithm-based assistance, including but not limited to (online) prediction models, automated radiographic analysis (i.e. segmentation, classification), diagnostic models, ML-based scoring systems, etc. Logistic and linear regressions are also considered ML. Other common ML algorithms include (deep) neural networks, random forests, decision trees, gradient boosting machines and naïve Bayes classifiers. The survey was developed by the authors based on prior, similar surveys carried out in a similar population [9, 10]. This report was constructed according to the Checklist for Reporting Results of Internet E-Surveys (CHERRIES) guidelines [8].

**Table 1:** Elements contained within the survey. Depending on the participants’ choice, nine or ten questions were displayed

Question	Response options	Response type
What is your primary subspecialty?	Spine; neurovascular; neuro-oncology; trauma; epilepsy, paediatric; peripheral nerve; neuro-intensive care; functional; other	Single choice; free text
What setting do you primarily practice in?	Academic hospital; non-academic hospital; private practice; other	Single choice; free text
What is your level of experience?	Resident; fellow; board-certified/attending; chairperson; medical student; other	Single choice; free text
What is your gender?	Male; female	Single choice
What age group are you in?	< 30 years; 30–40 years; 40–50 years; 50–60 years; > 60 years	Single choice
What country are you currently based in?	List	Single choice
**In your clinical practice, have you ever made use of machine learning?**	Yes, no	Single choice
**If yes:**		
What have you used machine learning for? Please select any of the applicable	Shared decision-making/patient information; outcome prediction; prediction of complications: interpretation/quantification of imaging; grading of disease severity; diagnosis; other	Multi-choice; free text
Please rate the importance of the following reasons for using machine learning from 1 to 4, based on your own clinical experience		
Improved preoperative surgical decision-making/treatment selection	1 (Not important) to 4 (Highly important)	Single choice
Improved anticipation of complications	1 (Not important) to 4 (Highly important)	Single choice
Objectivity in diagnosis/grading/risk assessment	1 (Not important) to 4 (Highly important)	Single choice
Improved shared decision-making/ patient information	1 (Not important) to 4 (Highly important)	Single choice
Time savings	1 (Not important) to 4 (Highly important)	Single choice
**If no:**		
Please rate the importance of the following reasons for not using machine learning from 1 to 4		
Not personally convinced of added value	1 (Not important) to 4 (Highly important)	Single choice
Lack of skilled resources (staff, equipment) to develop a model	1 (Not important) to 4 (Highly important)	Single choice
Lack of data (quantity/quality) to develop a model	1 (Not important) to 4 (Highly important)	Single choice
Limited time to implement ML in clinical practice	1 (Not important) to 4 (Highly important)	Single choice
Limited affordability	1 (Not important) to 4 (Highly important)	Single choice
Difficulties in deciding which processes may benefit most from application of ML algorithms	1 (Not important) to 4 (Highly important)	Single choice
Lack of ML models for my indications	1 (Not important) to 4 (Highly important)	Single choice
**In your research, have you ever made use of machine learning?**	Yes; No; I do not engage in medical research	Single choice

*ML*, machine learning

### Statistical analysis

Continuous variables are given as means ± standard deviations (SD), whereas categorical variables are reported as numbers (percentages). By use of multivariable logistic regression models, we identified independent predictors of adoption of ML algorithms into clinical practice and research, respectively. Countries were grouped by region (Europe/North America/Latin America/Asia and Pacific/Middle East/Africa) according to a previous worldwide survey by Härtl et al. [10], and response rates per region were calculated. Fisher’s exact test was applied to compare ML implementation rates among regions. The importance of reasons for use or non-use of ML was compared among regions using Kruskal-Wallis *H* tests. When calculating the ratio of respondents who had applied ML in research, we incorporated both respondents who had never used ML in their research as well as those who do not participate in medical research into the denominator. All analyses were carried out using R version 3.5.2 (the R Foundation for Statistical Computing, Vienna, Austria). A *p* ≤ 0.05 was considered statistically significant in two-sided tests.

## Results

### Response rate and respondent characteristics

A total of 7280 CNS/EANS members were sent the survey and 362 complete or incomplete answers were received for analysis. The descriptive data of respondents are provided in Table 2. The most represented age range was 30–40 (32.6%), and 89.2% of the answers were from male participants. Most of surveyed neurosurgeons were specialized in spine surgery (36.2%). As far as the work setting was concerned, more than two-thirds of the neurosurgeons were practicing in an academic hospital (67.4%), followed by non-academic hospitals (15.5%), private practice (15.5%) and other settings (1.7%). We also sought to describe the level of experience of the surveyed population. Participants were mostly board-certified/attending neurosurgeons (59.9%), while residents (19.1%), department chairs (11.3%), fellows (5.0%), medical students (2.2%) and others (2.5%) were less represented. Geographic distribution of the answers was skewed in favour of North America (69.1%) and Europe (18.8%), while less answers were received from surgeons from Asia and Pacific (4.1%), Latin America (5.0%), Middle East (2.5%) and Africa (0.6%), with only two responses for the latter region.

**Table 2:** Basic demographics of the respondent population

Characteristic	Value (*n* = 362)
Age groups, *n* (%) (years)	
< 30	28 (7.7)
30–40	118 (32.6)
40–50	96 (26.5)
50–60	61 (16.9)
> 60	59 (16.3)
Male gender, *n* (%)	323 (89.2)
Specialty, *n* (%)	
Spine	131 (36.2)
Neuro-oncology	64 (17.7)
Neurovascular	49 (13.5)
Paediatric	32 (8.8)
Functional	27 (7.5)
Trauma	16 (4.4)
Epilepsy	5 (1.4)
Neuro-intensive care	4 (1.1)
Skull base	1 (0.3)
Peripheral nerve	2 (0.6)
Other	31 (8.6)
Work setting, *n* (%)	
Academic hospital	244 (67.4)
Non-academic hospital	56 (15.5)
Private practice	56 (15.5)
Other	6 (1.7)
Level of experience, *n* (%)	
Board-certified/attending	217 (59.9)
Resident	69 (19.1)
Chairperson	41 (11.3)
Fellow	18 (5.0)
Medical student	8 (2.2)
Other	9 (2.5)
Geographic origin, *n* (%)	
North America	250 (69.1)
Europe	68 (18.8)
Asia and Pacific	15 (4.1)
Latin America	18 (5.0)
Middle East	9 (2.5)
Other	2 (0.6)
Use of machine learning in clinical practice, *n* (%)	103 (28.5)
Use of machine learning in research, *n* (%)	108 (31.1)

### Machine learning in clinical practice and research

**Fig. 1:**
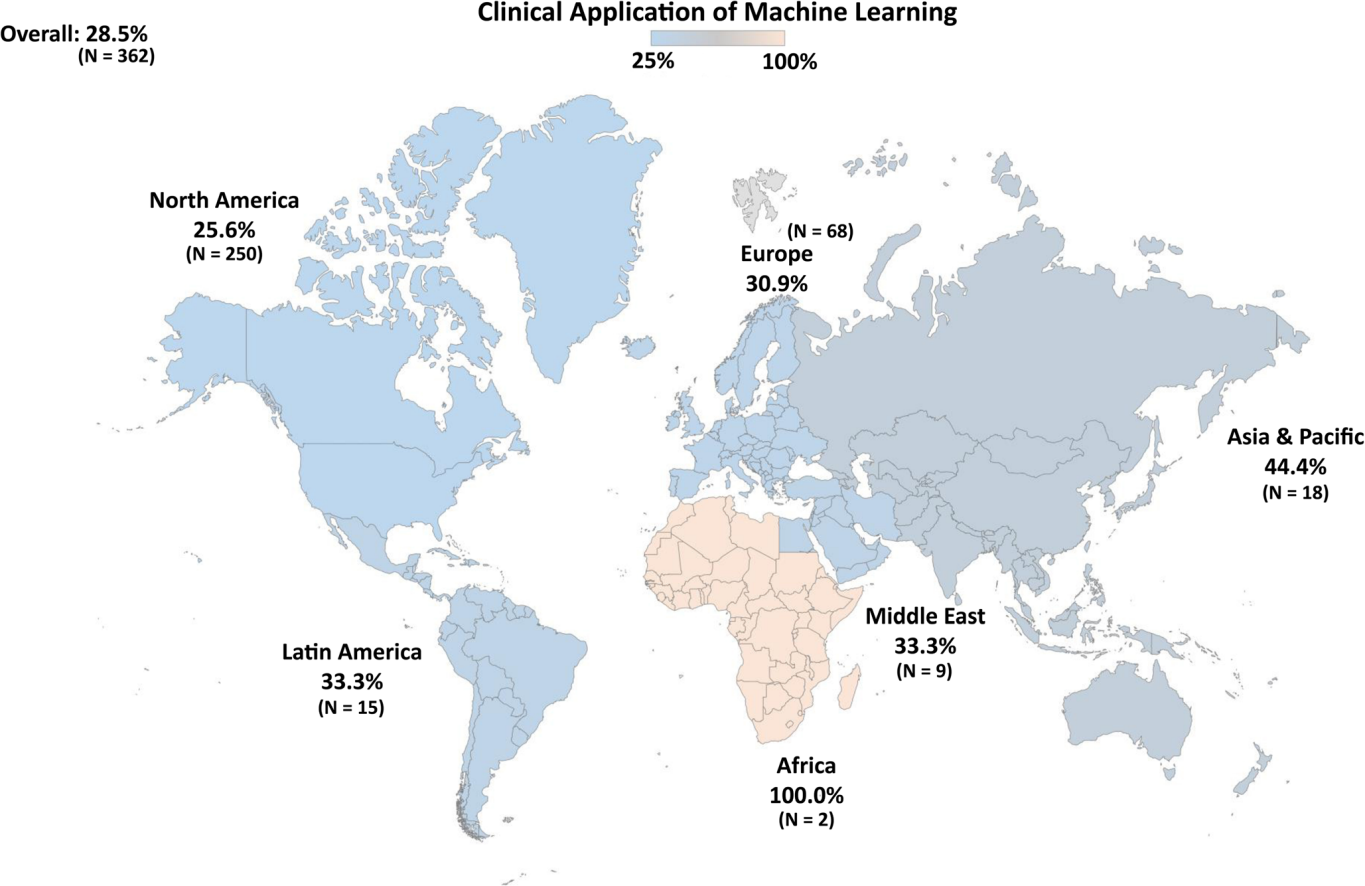
Proportions of neurosurgeons who report having used machine learning in their clinical practice among the 362 responders, stratified by region and plotted on a world map (Mercator projection)

A total of 28.5% and 31.1% of the surveyed population responded positively when asked about the use of ML in clinical practice and in clinical research, respectively. Concerning the use of ML in clinical practice, stratified by region (Table 3), adoption rates of ML were homogenously distributed (*p* = 0.125), with 25.6% for North America, 30.9% for Europe, 33.3% for Latin America and the Middle East, 44.4% for Asia and Pacific and 100% for Africa, albeit with only two responses. Figure 1 illustrates the worldwide clinical use of ML. We also asked respondents to list the kinds of applications that they employed ML for (Table 4). The most frequently reported uses of ML were for prediction of outcome (60.2%) and complications (51.5%), as well as to interpret or quantify medical imaging (50.5%). In addition, neurosurgeons applied ML to better inform their patients (38.8%), to grade disease severity (37.9%) and for diagnostic analytics (19.4%).

**Table 3:** Proportions of neurosurgeons who report having used machine learning in clinical practice or clinical research among the responders, stratified by region

Domain	Region							*p*
	Overall (*n* = 362)	North America (*n* = 250)	Europe (*n* = 68)	Latin America (*n* = 15)	Asia & Pacific (*n* = 18)	Middle East (*n* = 9)	Africa (*n* = 2)	
Clinical practice, *n* (%)	103/362 (28.5)	64 (25.6)	21 (30.9)	5 (33.3)	8 (44.4)	3 (33.3)	2 (100.0)	0.125
Clinical research, *n* (%)^a^	108/347 (31.1)	69/239 (28.9)	27/67 (40.3)	3/15 (20.0)	6/16 (37.5)	1/8 (12.5)	2/2 (100.0)	0.087

^a^While all responders answered the question on machine learning use in clinical practice, a subset did not answer the second question on application of machine learning in clinical research

**Table 4:** Reported applications of machine learning in clinical practice

Application	Frequency, *n* (%) (*n* = 103)
Outcome prediction	62 (60.2)
Prediction of complications	53 (51.5)
Interpretation/quantification of imaging	52 (50.5)
Shared decision-making/patient information	40 (38.8)
Grading of disease severity	39 (37.9)
Diagnosis	20 (19.4)

#### Predictors of machine learning use

Multivariate logistic regression analysis (Table 5) was used to investigate independent predictors of ML use in clinical practice and research. Our analysis revealed that none of the studied variables was associated with increased or decreased use of ML in clinical practice, demonstrating the wide and homogenous adoption of ML globally. On the other hand, surgeons specialized in neuro-oncology (odds ratio (OR) = 2.76, 95% confidence interval (CI) = 1.28 to 6.05, *p* = 0.010), functional neurosurgery (OR = 2.79, 95% CI = 1.03 to 7.47, *p* = 0.040), trauma (OR = 3.8, 95% CI = 1.44 to 10.02, *p* = 0.007) and epilepsy (OR = 3.8, 95% CI = 1.14 to 12.9, *p* = 0.030) were found to be significantly more likely to apply ML for research purposes with respect to the reference group. Also, when referenced to neurosurgeons working in academic hospitals, those working in non-academic centres (OR = 0.23, 95% CI = 0.08 to 0.57, *p* = 0.003) or in private practice (OR = 0.36, 95% CI = 0.14 to 0.85, *p* = 0.026) were significantly less likely to engage in ML-based research.

**Table 5:** Multivariable logistic regression models describing the relationship between covariates and adoption of machine learning into clinical practice and research, respectively

Variable	Clinical practice			Clinical research		
	OR	95% CI	*p* value	OR	95% CI	*p* value
Age group						
< 30	1.21	0.52 to 2.74	0.658	1.33	0.55 to 3.19	0.520
30–40	Reference	-	-	Reference	-	-
40–50	0.97	0.41 to 2.2	0.938	1.33	0.56 to 3.17	0.520
50–60	1.62	0.71 to 3.7	0.248	0.85	0.33 to 2.1	0.730
> 60	1.82	0.47 to 6.93	0.382	3.25	0.78 to 13.7	0.110
Male gender	0.97	0.43 to 2.27	0.935	2.19	0.89 to 5.94	0.100
Specialty						
Spine	Reference	-	-	Reference	-	-
Neuro-oncology	1.12	0.53 to 2.32	0.763	2.76	1.28 to 6.05	0.010*
Neurovascular	1.13	0.51 to 2.43	0.754	0.67	0.26 to 1.61	0.380
Paediatric	0.58	0.19 to 1.57	0.301	1.00	0.33 to 2.85	0.997
Functional	1.00	0.37 to 2.50	0.996	2.79	1.03 to 7.47	0.040*
Trauma	1.46	0.55 to 3.68	0.425	3.80	1.44 to 10.02	0.007*
Epilepsy	2.27	0.75 to 6.74	0.140	3.80	1.14 to 12.9	0.030*
Neuro-intensive care	NA	NA	0.991	NA	NA	0.990
Peripheral nerve	NA	NA	0.993	2.82	0.11 to 75.5	0.570
Skull base	1	0.05 to 8.93	0.997	2.01	0.09 to 20.12	0.480
Other	NA	NA	0.995	NA	NA	0.990
Setting						
Academic hospital	Reference	-	-	Reference	-	-
Non-academic hospital	0.67	0.30 to 1.43	0.315	0.23	0.08 to 0.57	0.003*
Private practice	0.59	0.26 to 1.28	0.195	0.36	0.14 to 0.85	0.026*
Other	1.11	0.13 to 6.89	0.915	NA	NA	0.990
Experience						
Board-certified/attending	Reference	-	-	Reference	-	-
Resident	1.40	0.56 to 3.6	0.458	1.14	0.44 to 3.00	0.790
Chairperson	1.58	0.68 to 3.58	0.279	2.03	0.80 to 5.17	0.130
Fellow	1.36	0.38 to 4.63	0.628	0.42	0.08 to 1.79	0.270
Medical student	1.18	0.17 to 7.37	0.860	1.10	0.17 to 8.04	0.920
Other	0.77	0.11 to 3.69	0.767	1.60	0.27 to 8.07	0.570
Geographic origin						
North America	Reference	-	-	Reference	-	-
Europe	1.12	0.57 to 2.16	0.738	1.32	0.65 to 2.63	0.440
Latin America	2.48	0.81 to 7.52	0.547	0.49	0.10 to 1.83	0.330
Asia and Pacific	1.43	0.41 to 4.46	0.106	1.42	0.35	0.630
Middle East	1.64	0.30 to 7.45	0.536	0.16	0.01 to 1.15	0.110
Other	NA	NA	0.992	NA	NA	0.999

*p ≤ 0.05

*OR*, odds ratio; *CI*, confidence interval

#### Attitudes towards machine learning in neurosurgery

The surveyed population was also asked to rate the importance of the factors that encouraged or prevented the application of ML in neurosurgical clinical practice (Table 6). Among those the surgeons adopting who had already adopted ML into their clinical practice, their most important reasons determining this choice were first improved preoperative surgical decision-making/treatment selection (3.27 ± 0.86), followed by objectivity in diagnosis/grading/risk assessment (3.22 ± 0.84), improved anticipation of complications (3.13 ± 0.92) and improved shared decision-making/patient information (3.07 ± 0.9), while less importance was given to potential time savings (2.62 ± 1.07). These attitudes towards the benefits of ML in clinical practice were compared among regions, with no significant differences between the regions apart from the anticipation of complications (*p* = 0.048).

**Table 6:** Tabulation of reasons for use and non-use of machine learning (ML) in clinical practice, stratified per region

	Region							
	All	North America	Europe	Asia and Pacific	Latin America	Middle East	Africa	*p* value
Reasons for use								
Improved preoperative surgical decision-making/treatment selection	3.27 ± 0.86	3.14 ± 0.92	3.57 ± 0.6	3.6 ± 0.55	3.5 ± 0.76	3 ± 1.41	3 ± 1.41	0.430
Improved anticipation of complications	3.13 ± 0.92	2.92 ± 0.96	3.57 ± 0.6	3.2 ± 0.84	3.62 ± 0.74	3 ± 1.41	3 ± 1.41	0.048*
Objectivity in diagnosis/grading/risk assessment	3.22 ± 0.84	3.25 ± 0.85	3.05 ± 0.74	3.4 ± 0.55	3.5 ± 0.76	3 ± 1.41	2.15 ± 2.12	0.680
Improved shared decision-making/patient information	3.07 ± 0.9	3.06 ± 0.97	3.14 ± 0.65	2.8 ± 0.84	3.38 ± 0.74	2.5 ± 0.71	2.5 ± 2.12	0.720
Time savings	2.62 ± 1.07	2.72 ± 1.03	2.29 ± 1.1	2.8 ± 1.1	2.5 ± 1.2	3 ± 1.41	2.5 ± 2.12	0.720
Reasons for non-use								
Not personally convinced of added value	2.04 ± 1.05	2.13 ± 1.05	1.77 ± 1.07	2 ± 0.94	1.56 ± 0.73	2.5 ± 1.22	NA	0.070
Lack of skilled resources (staff, equipment) to develop a model	3.11 ± 0.98	3.14 ± 0.97	3.02 ± 1.07	3.1 ± 1.1	2.78 ± 0.83	3.33 ± 0.82	NA	0.670
Lack of data (quantity/quality) to develop a model	2.67 ± 0.99	2.67 ± 0.99	2.72 ± 0.99	2.8 ± 0.92	1.78 ± 0.67	3.33 ± 0.82	NA	0.160
Limited time to implement ML in clinical practice	2.85 ± 0.96	2.85 ± 0.98	2.98 ± 0.94	2.9 ± 0.88	2.33 ± 0.71	2.33 ± 0.52	NA	0.160
Limited affordability	2.74 ± 1.08	2.77 ± 1.06	2.51 ± 1.16	2.5 ± 0.85	3.22 ± 1.09	3.33 ± 1.03	NA	0.034*
Difficulties in deciding which processes may benefit most from the application of ML algorithms	2.75 ± 0.96	2.77 ± 0.93	2.64 ± 1.11	2.6 ± 0.97	2.78 ± 0.83	3 ± 0.89	NA	0.900
Lack of ML models for my indications	2.84 ± 1	2.82 ± 0.99	2.79 ± 1.12	2.7 ± 0.67	3.44 ± 0.73	3.33 ± 0.82	NA	0.250

Continuous variables are presented as mean ± SD. The importance of reasons for use or non-use of robotics was compared among regions using Kruskal-Wallis H tests

*p ≤ 0.05

On the other hand, when asked to rate reasons for not using ML, lack of skilled resources (staff, equipment) to develop a model received the highest score (3.11 ± 0.98), followed by time limitations restricting ML application in clinical practice (2.85 ± 0.96), lack of available ML models for the indications of interest (2.84 ± 1), uncertainty concerning which processes may benefit most from application of ML algorithms (2.75 ± 0.96) and, less importantly, lack of data quantity/quality to develop a ML model (2.67 ± 0.99). The lack of personal conviction of the added value of ML scored last (2.04 ± 1.05). The only differences among regions were observed in terms of the affordability of ML applications—this reason for non-use of ML was rated significantly higher in the Middle East and Latin America (*p* = 0.034).

## Discussion

There exists no prior published data on the worldwide adoption of ML in neurosurgery. This global survey reached a diverse cohort of neurosurgeons at different levels of training. Our results indicate that ML has already quickly gained wide acceptance in the neurosurgical community, without notable heterogeneity in its global distribution. Almost a third of neurosurgeons reported having applied ML in either clinical practice or research, a value that exceeded expectations. Furthermore, the most common applications of ML in neurosurgery were for prediction of complications and outcomes, as well as to interpret or automatically quantify imaging. No predictors of clinical ML use were identified, again stressing that the availability and acceptance of readily developed ML tools are not bound by socio-demographic factors. On the other hand, among research-active neurosurgeons, some subspecialties as well as academic surgeons appear to apply ML more frequently for their research.

Our study is the first to our knowledge to provide a worldwide overview of the implementation of ML in neurosurgical clinical practice and research. To our surprise, almost a third of respondents stated making use of ML, and this was true for both clinical practice and research. Although this can be partially explained by response bias—with academic surgeons active in the EANS and CNS targeted and with a likely higher response rate to our survey among surgeons interested in ML—our results still indicate that ML is quickly becoming one of the foremost technologies in neurosurgical practice. Importantly, the heterogeneity in adoption rates among regions was relatively low, and adoption of ML into clinical practice was not apparently influenced by limitations in costs or socioeconomic status, as is the case with other less accessible technologies such as robotics [33, 35]. While the development of ML models can often be expensive and resource-intensive, the application of readily trained ML algorithms does not usually require especially high technological standards or expenses. Many ML applications are web-based [25]. For this reason, we expect that ML will increasingly enable enhanced diagnostic, prognostic and predictive analytics around the world, even in the most rural areas.

After controlling for potential confounding factors, we could not identify factors associated with increased or decreased use of ML in clinical practice. This again demonstrates how homogenously ML use seems to be distributed among the neurosurgical community. On the other hand, subspecialists in neuro-oncology, functional neurosurgery, trauma and epilepsy were significantly more likely to apply ML in their research. As expected, surgeons working in non-academic centres and private practice were less likely to engage in ML-based neurosurgical applications, consistent with the development of ML models currently being rather confined to academic institutions possessing the resources, protected time, expertise, extensive databases and computational power to create and distribute algorithms. However, it has to be considered that the development of e.g. ML-based prediction models has been massively eased by free software packages released by the major technology companies, which nowadays enable training of simple ML models on even the most basic notebooks. Still, the development of models may be limited by a lack of high-quality, structured datasets [24].

In fact, ML has already been broadly applied to several subspecialties in neurosurgery spanning from cranial [1, 7, 39], vascular [15, 32], spinal [5, 11, 13, 25, 31, 36] and radiosurgery, among others [23, 41]. Several examples of how ML outperforms traditional statistics and prognostic indexes commonly applied in the clinical practice are already available in the medical literature. For example, a recent study by van Niftrik et al. reported the use of a gradient boosting machine to predict early post-operative complications after intracranial tumour surgery [41]. The authors were able to show improved performance with respect to conventional statistical modelling based on logistic regression and interestingly observed that among the variables in their model, features that were not taken into account in the statistical model, such as histology, anatomical localization or surgical access in fact contributed strongly in the ML model [41]. Oermann et al. also showed that artificial neural networks performed better at 1-year survival prediction than more traditional models in patients with brain metastases treated with radiosurgery [22]. The same group also was able to show an improvement in predictions of arteriovenous malformation radiosurgery outcomes [23]. Staartjes et al. found that a deep learning approach was significantly better at predicting intraoperative cerebrospinal fluid leaks and gross total resection in pituitary surgery than logistic regression, while no predictors could be identified using traditional interferential statistics for the former outcome [34, 37].

In spinal neurosurgery, applications of ML have included prediction of outcome in patients with lumbar disc herniation and lumbar spinal stenosis [2, 31, 36], or to predict complications following elective adult spinal deformity procedures [14]. For example, Khor et al. developed a prediction model from a state-wide database to predict clinically relevant improvement after lumbar spinal fusion and integrated their model into a freely available web app, which was then externally validated [13, 25].. Again, this shows that while it may be resource-intensive to develop such models, they can be rolled out to clinicians and patients around the world for free using simple interfaces.

Radiological applications are ideally suited to machine learning algorithms given the magnitude and complexity of data extractable from examinations such as CT and MRI scans. Interestingly, ML models can establish a hidden relationship between deep radiological features (“radiomics”) and outcomes of the pathology of interest. Lao et al., for example, were able to stratify patients into different prognostic subgroups based on radiomic features [17]. Similarly, it has been shown that it is possible to identify IDH mutation status in gliomas from radiomic features alone [4]. Finally, more extravagant applications of ML in neuroradiology include e.g. the generation of synthetic CT images—practically indistinguishable from actual CTs—from cranial MRI [6, 42].

Despite these positive results, still many present and future potential ML applications remain unknown to the majority of neurosurgical specialists. Our study determined that the factors deterring the use of ML were, in decreasing order, lack of skilled resources (staff, equipment) to develop a model, time limitations restricting ML application in clinical practice, lack of ML models for the indications of interest, uncertainty concerning which processes may benefit most from the application of ML algorithms, as well as—less importantly—lack of data to develop a model, and lack of personal convincement of the added value of this new technology.

Our results warrant some considerations. First, once a ML model with clinical relevance is developed and after it has been externally validated [25], the focus has to shift on making it easy to implement and widely available in clinical practice. Web-based apps that are clinician- or patient-friendly are ideal [12, 13, 25]. Second, while a large proportion of neurosurgeons may already be applying ML in their clinical practice, it is important to foster ML literacy in the neurosurgical community. As with randomized studies forming the basis of evidence-based practice, clinicians should be able to make an informed decision as to which ML models published are likely valid and have applied good methodology, and which ones should probably not be trusted in clinical practice. Lastly, ML relies on the availability of “big data” to be exploited for algorithm training and validation subsequently [21, 24]. A wide and complete collection of patient data in the sense of population-based databases enables more representative ML models. Integrated databases with automated comprehensive data collection that are necessary for such applications are currently few and far between, preventing the development of highly generalizable models [20, 21, 24, 27].

## Limitations

Survey-based studies, while able to provide important insights, have inherent limits because of several potential biases. During survey distribution, selection and response bias are frequent. Time constraints on responders may have limited their ability to answer with maximal accuracy, and in fact concerning the adoption of ML into clinical research, we obtained several incomplete or blank answers. The data is mostly based on subjective impressions of surgeons. Knowing this, bias could arise from the fact that surgeons who are more exposed to neurosurgical ML can value it more positively than those who do not routinely make use of it, and vice-versa. However, the reasons for advantages and disadvantages were specifically captured separately for users and non-users. Additionally, the relative percentage of geographic regions was skewed in favour of western countries, limiting the sensitivity of our survey for what concerns regions such as Asia and Pacific, South America and in particular Africa with only two responses.

## Conclusions

This study provides a first global overview of the adoption of ML into neurosurgical practice. Machine learning has the potential to improve diagnostic work-up and neurosurgical decision-making by shedding light on radiological interpretation, surgical outcome and complication prediction and as a consequence patients’ quality of life and surgical satisfaction. A relevant proportion of neurosurgeons appears to already have adopted ML into their clinical practice in some form. The homogenous distribution of ML users in neurosurgery is a testimony to the accessibility of readily developed ML algorithms, even in low-resource settings. Still, many structural issues need to be addressed in order for ML to achieve its full potential in neurosurgery. These include easy-to-access resources for surgeons and patients; prospective-integrated data collection systems to allow model development; and surgeon education on ML, all of which can add to the rapid development of ML in neurosurgery while ensuring high quality of the introduced tools and their correct application. Best practice recommendations, external validation and sound methodology are necessary for any ML tool before its application in our high-stakes clinical practice. Furthermore, future trials may be conducted to assess the real clinical impact—and any changes in decision-making—that may be caused by ML algorithms in neurosurgery.
